# Value of Apparent Diffusion Coefficient Histogram Analysis in the Differential Diagnosis of Nasopharyngeal Lymphoma and Nasopharyngeal Carcinoma Based on Readout-Segmented Diffusion-Weighted Imaging

**DOI:** 10.3389/fonc.2021.632796

**Published:** 2021-03-12

**Authors:** Chengru Song, Peng Cheng, Jingliang Cheng, Yong Zhang, Shanshan Xie

**Affiliations:** ^1^Department of MRI, The First Affiliated Hospital of Zhengzhou University, Zhengzhou, China; ^2^Department of radiotherapy, Henan Provincial People’s Hospital, Zhengzhou, China

**Keywords:** nasopharyngeal carcinoma, lymphoma, magnetic resonance imaging, diffusion magnetic resonance imaging, histogram

## Abstract

**Background:**

This study aims to explore the utility of whole-lesion apparent diffusion coefficient (ADC) histogram analysis for differentiating nasopharyngeal lymphoma (NPL) from nasopharyngeal carcinoma (NPC) following readout-segmented echo-planar diffusion-weighted imaging (RESOLVE sequence).

**Methods:**

Thirty-eight patients with NPL and 62 patients with NPC, who received routine head-and-neck MRI and RESOLVE (b-value: 0 and 1,000 s/mm^2^) examinations, were retrospectively evaluated as derivation cohort (February 2015 to August 2018); another 23 patients were analyzed as validation cohort (September 2018 to December 2019). The RESOLVE data were obtained from the MAGNETOM Skyra 3T MR system (Siemens Healthcare, Erlangen, Germany). Fifteen parameters derived from the whole-lesion histogram analysis (ADC_mean_, variance, skewness, kurtosis, ADC_1_, ADC_10_, ADC_20_, ADC_30_, ADC_40_, ADC_50_, ADC_60_, ADC_70_, ADC_80_, ADC_90_, and ADC_99_) were calculated for each patient. Then, statistical analyses were performed between the two groups to determine the statistical significance of each histogram parameter. A receiver operating characteristic curve (ROC) analysis was conducted to assess the diagnostic performance of each histogram parameter for distinguishing NPL from NPC and further tested in the validation cohort; calibration of the selected parameter was tested with Hosmer–Lemeshow test.

**Results:**

NPL exhibited significantly lower ADC_mean_, variance, ADC_1_, ADC_10_, ADC_20_, ADC_30_, ADC_40_, ADC_50_, ADC_60_, ADC_70_, ADC_80_, ADC_90_ and ADC_99_, when compared to NPC (all, *P* < 0.05), while no significant differences were found on skewness and kurtosis. Furthermore, ADC_99_ revealed the highest diagnostic efficiency, followed by ADC_10_ and ADC_20_. Optimal diagnostic performance (AUC = 0.790, sensitivity = 91.9%, and specificity = 63.2%) could be achieved when setting ADC_99_ = 1,485.0 × 10^−6^ mm^2^/s as the threshold value. The predictive performance was maintained in the validation cohort (AUC = 0.817, sensitivity = 94.6%, and specificity = 56.2%)

**Conclusion:**

Whole-lesion ADC histograms based on RESOLVE are effective in differentiating NPC from NPL.

## Background

Nasopharyngeal carcinoma (NPC) and nasopharyngeal lymphoma (NPL) are two of the most common types of nasopharyngeal malignancies. The differential diagnosis is generally difficult, since NPC and NPL always have parallel clinical manifestation, such as nasal obstruction, epistaxis, and headache. Although NPL is rarely seen, the occurrence rate of lymphoma has continuously increased of late years. These two tumors significantly differ from each other in the aspect of biological behavior, prognosis, and therapeutic methods. Hence, a precise diagnosis is essential to optimize individual therapeutic regimens. Therefore, it is of significant clinical significance to differentiate NPC from NPL in the early phase.

Magnetic resonance imaging (MRI) and computed tomography (CT) are the prime non-invasive imaging methods applied to the diagnosis of nasopharyngeal tumors. Traditional MRI would, without doubt, provide a good description of the tumor and the relationship of anatomical structures. However, NPC and NPL usually share parallel imaging features on routine pre- or post-contrast scans (1), thereby leading to poor differentiating diagnostic accuracy.

To date, the availability of diffusion-weighted imaging (DWI) has been widely confirmed in head and neck tumors, such as head and neck lesions (2, 3), and maxillofacial (4), orbital (5), or sinonasal neoplasms (6). However, very few studies have focused on tumors in the nasopharynx, especially differentiating NPC from NPL (7, 8). Moreover, it is noteworthy that readout-segmented echo-planar diffusion-weighted imaging (RESOLVE sequence) can promote image quality in the area of head and neck, when compared to routine DWI, which uses the single-shot echo-planar imaging (SS-EPI) technique, reducing T_2_* blurring and susceptibility artifacts by reduce echo-spacing (9, 10). The apparent diffusion coefficient (ADC) maps acquired from the RESOLVE-DWI own a higher resolution and signal-to-noise ratio compared to routine DWI, thereby significantly improved the diagnostic reliability and efficiency of the ADC value. In addition, these ADC values are usually calculated by drawing a single region of interest (ROI) over the solid part of the tumor or over the maximum area of the tumor. However, this freehand procedure is often not sufficient sensitive to unconspicuous changes.

As an up-to-date technique of image processing, the whole-lesion histogram features of ADC maps based on voxel distribution can eliminate sampling bias and improve the reproducibility of the quantitative analysis. Furthermore, the assessment of the tumor heterogeneity can be improved with the application of histograms, which has been verified in previous studies of Head and Neck Squamous Cell Carcinoma (11, 12) and thyroid cancer (13, 14). Histogram parameters, including mean value, variance, kurtosis, skewness, and percentiles, are used to quantitatively describe the distributions of tumor biomarkers. With the continuous development in both signal processing methods and high-resolution MRI, the histogram analysis of MRI is being used in the characterization of tumors cumulatively, such as endometrial cancer (15), rectal cancer (16), lymph nodes in the head and neck region (17), glioma (18), posterior fossa tumors (19), *etc*. However, the application of RESOLVE-DWI or histograms in nasopharynx tumors was rarely reported.

The present study attempted to explore the utility of whole-lesion ADC histogram analysis for differentiating NPL from NPC following RESOLVE imaging.

## Methods

### Patients

The present study was approved by the Review Committee of the First Affiliated Hospital of Zhengzhou University (No:2015010039). For derivation cohort, the data obtained from patients with pathologically proved NPC or NPL in our hospital from February 2015 to August 2018 were analyzed; for validation cohort, the same set of data were collected for patients admitted from September 2018 to December 2019. The inclusion criteria were as below: (1) patients without previous treatment, surgery, or biopsy, and (2) patients who successfully received RESOLVE-DWI and routine MRI of the nasopharynx. The exclusion criteria were as below: (1) patients with obvious susceptibility or motion artifacts and (2) patients with small tumor volume (anteroposterior diameter <1 cm) that could lead to difficulties in the process of image analysis.

### MRI Examination

MRI examinations were achieved on a 3.0 T MR scanner (Magnetom Skyra, Siemens Healthcare, Erlangen, Germany) with a head and neck coil (integrated 20-channel). The pre-contrast scan protocols were listed below: (1) axial T1 weighted spin echo images: repetition time (TR) = 600 ms, echo time (TE) = 9 ms, section thickness = 4 mm, and intersection gap = 1 mm; (2) fat suppressed axial/sagittal/coronal T2 weighted spin echo images: TR = 4300 ms, TE = 111 ms, section thickness = 4 mm, and intersection gap = 1 mm; (3) DWI using readout-segmented echo-planar imaging, parallel imaging, and two-dimensional navigator-based reacquisition: five readout segments, echo spacing = 0.34 ms, b value = 0 and 1,000 s/mm^2^, section thickness = 4 mm, intersection gap = 1 mm, FOV = 178 × 178 mm, matrix = 178 × 178, TR = 3900 ms, TE = 64 ms; (4) fat suppressed axial/sagittal/coronal T1 weighted contrast scans: TR = 884 ms, TE = 6.8 ms, section thickness = 4 mm, and intersection gap = 1 mm. Gadolinium diethylenetriamine pentaacetic acid (Gd-DTPA, Magnevist, Schering, Berlin, Germany) was intravenously injected at a rate of 2 ml/s (total dose, 0.1 mmol/kg of body weight), followed by a 20-ml saline flush.

### Image Analysis

The general MR features including lesion homogeneity, symmetry, volume, and enhancement intensity were analyzed and compared between NPL and NPC patients. The lesion was considered homogeneous as the lesion signal intensity was within 10% of median values on 90% of voxels within the lesion. The degree of enhancement was defined as: High-enhancement that was equal or higher than the nasal mucosa. Low-enhancement that was equal or lower than adjacent muscles. Intermediate- degree of enhancement was between the adjacent muscles and nasal mucosa.

The ADC map was automatically reconstructed after the scan of RESOLVE. All ADC histogram data were post-processed offline using an Image processing software (ImageJ, version 1.47; https://imagej.nih.gov/ij/) by two experienced radiologists. In order to maintain the consistency among different cases, the window width and window level were adjusted to 2,560 and 1,280 respectively in advance. Then, the region of interest (ROI) including the hemorrhagic, cystic, or necrosis portion was manually drawn around the whole tumor margin (FOV 178×178 mm, matrix 178 × 178; ADC FOV 178 × 178mm, matrix 178 × 178) with reference to the pre- and post-contrast MRI and DWI image. The high-signal intensity areas were considered as tumor tissues (20); boundaries were drawn along these areas to ensure that the entire lesion was included. For evaluating the inter-reader variability of ROIs, each of the two readers extracted mean ROI value from the source images that they have not previously seen; the software will calculate the mean values after the delineation of ROIs. The inter-reader variability was analyzed using the coefficient of variation (CV) for mean ROIs between the two readers. CV was calculated as SD divided by the mean. Subsequently, the histogram and frequency distribution table of each section were automatically generated. The frequency distribution table of all sections was imported into the Excel software (Version 1909, Microsoft Corp, Chicago, IL, USA) for the summary, and the frequency distribution table of the whole tumor was obtained. SPSS (Version 25.0 Armonk, NY: IBM Corp) was used for the histogram analysis, and 15 ADC histogram parameters (ADC_mean_, variance, skewness, kurtosis, ADC_1_, ADC_10_, ADC_20_, ADC_30_, ADC_40_, ADC_50_, ADC_60_, ADC_70_, ADC_80_, ADC_90_, and ADC_99_) were calculated. The ADC histogram was plotted with the value of the ADC map on the X-axis, while the Y-axis was expressed as the frequency of each ADC value.

**Figure 1:**
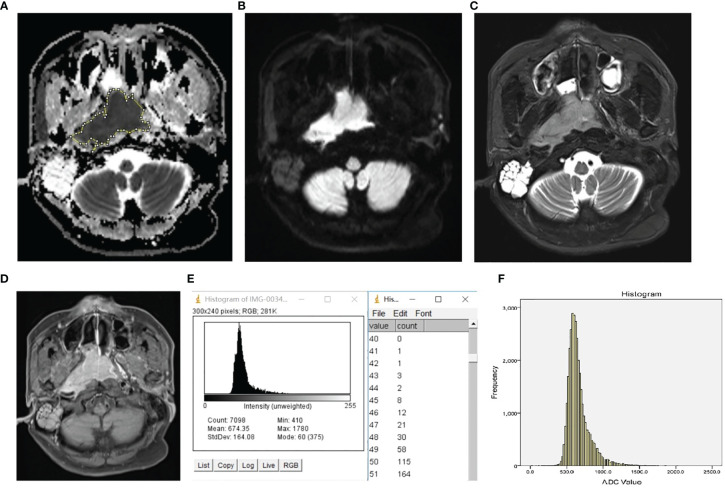
Representative ROI selection schematic illustration and histogram of NPL. The ROI was manually drawn around the whole tumor margin on each section of the ADC map **(A)** with reference to DW image **(B)**, T2 weighted image with fat suppression **(C)**, and T1 weighted post-contrast image with fat suppression **(D)**. The histogram and frequency distribution table of each section **(E)** were automatically generated. After adding up the frequency distribution data of all sections, the whole-tumor histogram **(F)** was generated by SPSS.

The representative images, which illustrate how the ROI was placed, and the histograms are presented in **Figure 1**.

ADC_mean_ is the average of all levels of the ADC maps within the whole-tumor ROI (21). ADCn is the point at which the n% of the voxel values that form the histogram is found to the left (21). Calculation of ADCmean, ADCn, skewness, and variation were performed according to the methodology of a previous study (21).

### Statistical Analysis

The histogram data of all the 15 parameters were evaluated for normality using the Kolmogorov–Smirnov test and test of variance homogeneity. Parameters that conformed to the normal distribution were expressed as mean ± standard deviation, while two-independent sample *t*-test was performed for comparisons between two groups. The other parameter values were expressed as median ± interquartile range, and the Mann–Whitney U test was used for comparison. Receiver operating characteristic (ROC) curve analysis was used to investigate the diagnostic ability of significant parameters for discrimination; calibration was assessed using Hosmer–Lemeshow test with associated *P* value. The cut-off value was calculated using the maximum of the Youden index (Youden index = sensitivity + specificity − 1). All statistical analysis was performed using SPSS. A probability of *P <*0.05 was considered statistically significant.

## Results

For derivation cohort, 62 patients with NPC (54 non-keratinizing and eight keratinizing) and 38 patients with lymphoma (18 patients with NK/T lymphoma, 11 patients with diffuse large B-cell lymphoma, two patients with classic mantle cell lymphoma, two patients with Burkitt lymphoma, two patients with angioimmunoblastic T-cell lymphoma, one patient with peripheral T-cell lymphoma, one patient with anaplastic large cell lymphoma, and one patient with T-lymphoblastic lymphoma) were included in the present study. In the NPC group, 51 male and 11 female patients were included, and their mean age was 48.97 years old, which ranged within 9–85 years old. In the NPL group, 27 male and 11 female patients were included, and their mean age was 46.16 years old, which ranged within 6–76 years old. Baseline, histological and ADC values of the patients were presented in **Table 1**. For validation cohort, 16 patients (12 non-keratinizing and four keratinizing) with NPC and seven patients (six patients with NK/T lymphoma and one patient with diffuse large B cell lymphoma) with NPL were included; there were 18 male and five females, and their mean age was 48 years old.

**Table 1:** Histologic subtypes of patients with NPL and NPC.

Subtype	No. of Patients			Age Range (y)	Mean Age (y)	ADC Range(10^-6^ mm^2^/second)	Mean ADC (10^-6^ mm^2^/second)± Standard Deviation
	Male Female Total						
NPL	11(28.9%)^a^				46.2^b^		
NK/T lymphoma	15	3	18	10~70	44.2	630.01~1064.02	798.51±123.25
Diffuse large B-cell lymphoma	6	5	11	27~76	57.0	556.03~1055.43	746.83±155.02
Burkitt lymphoma	2	0	2	9~15	12.0	464.84~517.81	491.35±37.54
Angioimmunoblastic T-cell lymphoma	1	1	2	61	61.0	632.15~1030.26	831.15±281.52
Classic mantle cell lymphoma	1	1	2	70~75	72.5	513.43~638.85	576.18±88.73
T-lymphoblastic lymphoma	1	0	1	6	6	533.21	533.24
Anaplastic large cell lymphoma	0	1	1	10	10	873.93	873.92
Peripheral T-cell lymphoma	1	0	1	25	25	868.14	868.16
NPC	11(17.7%)				49.0		
Non-keratinizing	45	9	54	9~85	49.1	695.32~1100.93	866.82±80.25
Kratinizing	6	2	8	18~63	48.9	793.54~1153.01	904.8±109.51

a Difference of female proportion were not significant when compared between NPL and NPC patients: 28.9% to 17.7%, P = 0.21.

b Mean age were not significant between two groups:46.2 years to 49.0 years, P = 0.16.

ADC, apparent diffusion coefficient; NPC, nasopharyngeal carcinoma; NPL, nasopharyngeal lymphoma.

The statistical results revealed that only three parameters (ADC_mean_, ADC_70_, and ADC_80_) conformed to the normal distribution. Thus, for these three parameters, two-independent sample t-test was used for comparison, while Mann–Whitney U-test was used to evaluate the other parameters. Coefficient of variation of ROIs between the two readers was 6.55%, thus the inter-reader variability of ROIs was considered small.

As depicted in **Table 1**, there were no statistical difference between patients with NPL or NPC in consideration of mean age (46.2 years to 49.0 years, *P = 0.16*) or sex proportion (as for female, 28.9 to 17.7%, *P = 0.21*).

The features of MR image between patients with NPL and NPC were recorded in **Table 2**, When compared to NPC patients, lesions of NPL patients tend to be more symmetric (44.7 to 22.6%, *P* = 0.02) and homogenous on T1WI (92.1 to 72.6%, *P* = 0.018), T2WI (57.9 to 32.3%, *P* = 0.012), while the distribution of the degrees of enhancement was not significantly different between the two groups (*P* = 0.120). **Table 3** summarizes the detailed comparison of ADC histogram parameters between NPL and NPC. The 13 parameters (ADC_mean_, variance, ADC_1_, ADC_10_, ADC_20_, ADC_30_, ADC_40_, ADC_50_, ADC_60_, ADC_70_, ADC_80_, ADC_90_ and ADC_99_) between these two groups were statistically different (all, *P* < 0.05). In addition, the NPL group had lower values for the above 13 parameters, when compared to those in the NPC group. However, no significant differences were found on the other two parameters (kurtosis and skewness).

**Table 2:** Features of MR images between patients with NPL or NPC.

Image parameter	NPL	NPC	P value
Lesion symmetry	17/38 (44.7%)	14/62 (22.6%)	0.020
Lesion homogeneity T1WI T2WI Gd-T1WI	35/38 (92.1%) 22/38 (57.9%) 23/38 (60.5%)	45/62 (72.6%) 20/62 (32.3%) 28/62 (45.2%)	0.018 0.012 0.136
Degree of enhancement High Intermediate Low	16/38 (42.1%) 21/38 (55.3%) 1/38 (2.6%)	17/62 (27.4%) 45/62 (72.6%) 0/62 (0.0%)	0.120

Gd-T1WI, gadolinium-enhanced T1-weighted image; NPC, nasopharyngeal carcinoma; NPL, primary nasopharyngeal lymphoma; T1WI, T1-weighted image; T2WI, T2-weighted image.

**Table 3:** Differences of ADC Histogram Parameters between NPL and NPC.

Parameters	NPL	NPC	*t* / *Z* value	*P* value
ADC_mean_	754.2 ± 157.0*	871.7 ± 84.4*	*t* = 4.249	<0.001
variance	444.52 ± 335.18	519.22 ± 185.36	*Z* = 2.251	0.024
skewness	1.33 ± 1.18	1.39 ± 0.38*	*Z* = 0.156	0.876
kurtosis	2.52 ± 5.41	3.05 ± 2.53	*Z* = 0.256	0.798
ADC_1_	412.6 ± 123.0*	500.0 ± 90.0	*Z* = 3.451	0.001
ADC_10_	537.6 ± 116.8*	640.0 ± 70.0	*Z* = 4.368	<0.001
ADC_20_	585.8 ± 124.6*	690.0 ± 72.5	*Z* = 4.194	<0.001
ADC_30_	625.5 ± 133.6*	735.0 ± 80.0	*Z* = 4.002	<0.001
ADC_40_	663.9 ± 142.0*	775.0 ± 100.0	*Z* = 3.852	<0.001
ADC_50_	705.3 ± 151.4*	810.0 ± 100.0	*Z* = 3.706	<0.001
ADC_60_	754.2 ± 163.6*	860.0 ± 120.0	*Z* = 3.645	<0.001
ADC_70_	816.8 ± 180.6*	932.0 ± 100.2*	*t* = 3.605	0.001
ADC_80_	901.1 ± 203.3*	1026.0 ± 111.8*	*t* = 3.480	0.001
ADC_90_	1039.7 ± 231.4*	1146.5 ± 152.5	*Z* = 3.918	<0.001
ADC_99_	1420.0 ± 265.0	1622.4 ± 252.0	*Z* = 4.858	<0.001

*indicate the data are consistent with normal distribution and are presented as mean ± SD; the other data are presented as median ± IQR.

Unit of ADCs are 10^-6^ mm^2^/second.

ADC, apparent diffusion coefficient; ADCn, nth percentile value of accumulative ADC histogram; IQR, Interquartile Range; NPL, nasopharyngeal lymphoma; NPC, nasopharyngeal carcinoma; SD, standard deviation.

**Table 4** summarizes the diagnostic performance of significant ADC histogram parameters in differentiating NPL from NPC. The best diagnostic performance was achieved at a threshold of ADC_99_ = 1,485.0 × 10^−6^ mm^2^/s (AUC = 0.790, sensitivity = 91.9%, specificity = 63.2%, Youden index = 0.551), followed by ADC_10_ = 545.0 × 10^−6^ mm^2^/s (AUC = 0.761, sensitivity = 95.2%, specificity = 52.6%) and ADC_20_ = 620.0 × 10^−6^ mm^2^/s (AUC = 0.750, sensitivity = 83.9%, specificity = 63.2%). The difference of the AUC between ADC_99_ and ADC_10_ was significant (*P* = 0.035).

**Table 4:** ROC Analysis of ADC Histogram Parameters for Discriminating NPL from NPC.

Parameters	AUC	Cutoff value	Sensitivity	Specificity	Youden Index
ADC_mean_	0.747	743.9	96.8%	55.3%	0.521
variance	0.635	370.94	95.2%	36.8%	0.320
ADC_1_	0.706	445.0	77.4%	63.2%	0.406
ADC_10_	0.761^*^	545.0	95.2%	52.6%	0.478
ADC_20_	0.750	620.0	83.9%	63.2%	0.471
ADC_30_	0.739	650.0	85.5%	60.5%	0.460
ADC_40_	0.730	675.0	88.7%	57.9%	0.466
ADC_50_	0.721	715.0	88.7%	57.9%	0.466
ADC_60_	0.718	765.0	87.1%	60.5%	0.476
ADC_70_	0.713	825.0	88.7%	60.5%	0.492
ADC_80_	0.718	895.0	90.3%	55.3%	0.456
ADC_90_	0.734	1065.0	85.5%	63.2%	0.487
ADC_99_	0.790^*^	1485.0	91.9%	63.2%	0.551

Unit of ADCs are 10^-6^ mm^2^/second.

*The difference of the AUC between ADC_99_ and ADC_10_ was significant (P = 0.035).

ADC, apparent diffusion coefficient; ADCn, nth percentile value of accumulative ADC histogram; AUC, area under the ROC curve.

**Figure 2:**
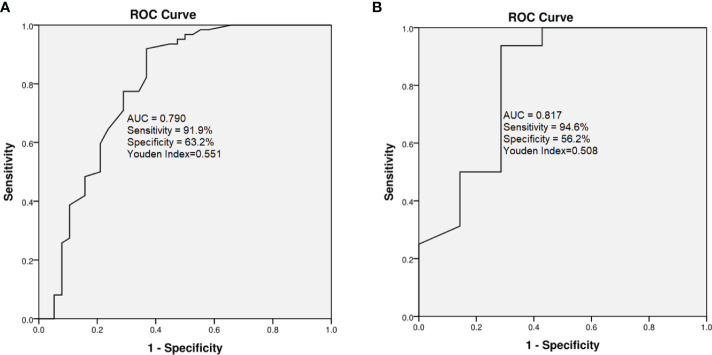
Comparison of ROC curves for ADC_99_ in derivation **(A)** and validation cohorts **(B)**. In derivation cohort, AUC = 0.790, sensitivity = 91.9%, specificity = 63.2%, Youden Index = 0.551; in the validation cohort, AUC 0.817, sensitivity = 94.6% and specificity = 56.2%, Youden Index = 0.508.

The validation cohort was used to verify the predictive performance and calibration of ADC_99_; ROC curve tested in this cohort showed an AUC of 0.817 (sensitivity = 94.6% and specificity = 56.2%, Youden index = 0.508, detailed in **Figure 2**), with a Hosmer–Lemeshow chi-square of 4.3 (*P* = 0.138).

## Discussion

The aim of this study was to determine the role of whole-lesion ADC histogram analysis for differentiating NPL from NPC based on RESOLVE imaging. The results demonstrated that among the 15 histogram parameters, a total of 13 parameters significantly differed between the NPL and NPC groups, which indicate that in addition to the normal MR feature that differs between the two groups (**Table 2**), the whole-lesion histogram analysis of ADC maps can also help to efficiently differentiate NPL from NPC and demonstrate the tumors’ heterogeneity. Furthermore, the ROC curve analysis of parameters with statistical difference revealed that the ADC_99_ might be the most promising parameter for the differentiation work and further test in validation cohort showed good performance and calibration.

As a functional imaging technique, DWI, which can measure the mobility of water molecules in tissues, has been applied to distinguish NPL from NPC (1, 7, 8). In previous studies (1, 7, 8), ADC values were most widely measured by the single-shot echo-planar imaging (SS-EPI) DWI sequence. However, since SS-EPI is susceptible to magnetic susceptibility artifacts, its image quality has not always been satisfying (9). Furthermore, few studies (22, 23) have reported the application of RESOLVE-DWI for nasopharynx tumors, as well as the discrimination of NPL and NPC. In the present study, the ADC maps used for the histogram analysis were obtained using the RESOLVE sequence. RESOLVE is a novel technique that uses two-dimensional navigator-based reacquisition to perform a non-linear phase correction and control the real-time reacquisition of unusable data that cannot be corrected (24). Furthermore, RESOLVE has been used to acquire high resolution DWI images, and the use of parallel imaging allows for it to have suitable scan time for clinical routine applications (25, 26). Zhao et al. (24) reported that RESOLVE prominently improves image quality in evaluating lesions in nasal sinus, and offers more precise ADC values, when compared to SS-EPI. Meanwhile, compared to the majority of the previous studies, which have been mostly based on selected ROIs drawn on the tumor’s solid part for analysis, whole-lesion ADC histogram analysis is a more impersonal technique, which provides measurable information on the heterogeneity and tissue characteristics of the whole tumor (27).

In the present study, the ADCmean of NPL was much lower than that of NPC, which was in correspondence with previous similar studies (1, 7). In addition, the other ADC parameters (ADC_1_, ADC_10_, ADC_20_, ADC_30_, ADC_40_, ADC_50_, ADC_60_, ADC_70_, ADC_80_, ADC_90_ and ADC_99_) of NPL were also lower than that of NPL. Depending mainly on the composition of the extra-cellular matrix and cell density, the ADC value has been generally considered to be positively correlated with the volume of the extravascular extracellular space and inversely correlated with tissue cellularity (13, 14). Lymphomas often show smaller ADCs, since lymphoma cells have relatively high nuclear-to-cytoplasm ratios and are densely packed throughout lesions (17, 28, 29).

Variance, skewness, and kurtosis have been indicated as promising biomarkers relevant to tumor heterogeneity. Variance represents the dispersion of the histogram. In the present study, NPC had a higher variance value than NPL, which may indicate that the signals of NPC lesions are more heterogeneous, when compared to NPL, in ADC maps, and that the ADC value’s distribution is more discrete. Therefore, this suggests that the tumor heterogeneity of NPC is higher. Previous observations (17, 29) have implied that NPC has a higher rate of necrosis and cystization, and more significant tumor heterogeneity, which is in line with the present results. However, no significant difference was found on skewness and kurtosis. Hence, further studies with larger sample sizes or more advanced texture analysis methods are needed.

The diagnostic superiority of ADC values has been reported in various tumors (30, 31). The study conducted by Guan et al. revealed that ADC_90_ was the strongest predictive indicator for differentiating tumors from normal cervical tissues (30). In the present study, ADC_99_ also performed better in the discrimination between NPL and NPC. This might be associated with the inclusion of necrosis and cystic areas, which have a large fluctuation of ADC values. However, ADC_10_, ADC_20_, and ADC_mean_ also revealed good differentiating performance.

**Figure 3:**
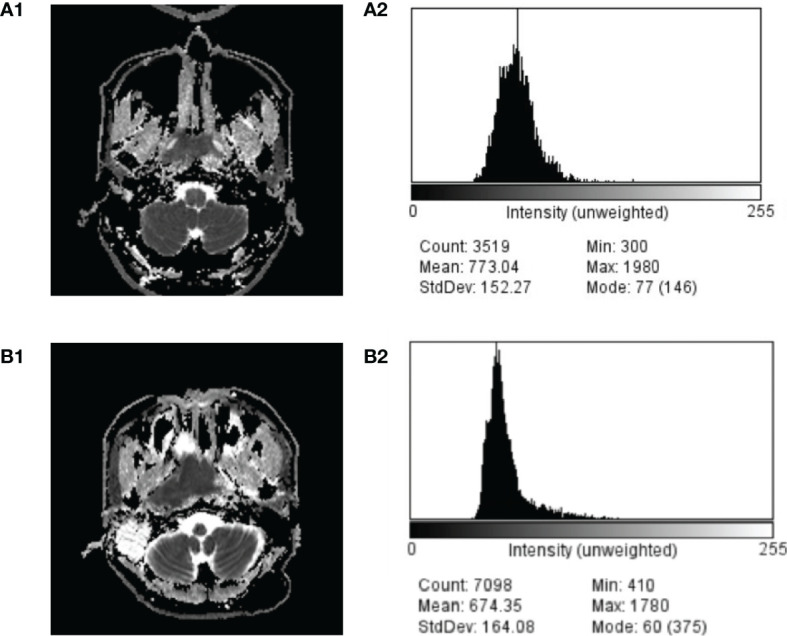
Comparison of ADC image and ADC value distribution of a case of NPC **(A1, A2)** and NPL **(B1, B2)**.

In our study, the difference of ADCmean was significant between NPL and NPC cases, and the AUC of ADC_99_ was slightly higher than those of ADCmeans. Thus, ADC_99_ didn’t provide much advance in terms of differential diagnosis between NPL and NPC. However, previous studies (32) of the application of ADC histogram in the differential diagnosis of various brain tumors had described the advantage of this method over ADC values and means alone, for the range of different brain tumors usually overlap a lot. In our cohort, the overlap of ADC range also existed between different histological subtypes of NPL and NPCs (**Table 1**). **Figure 3** depicted the ADC image and ADC value distributions of a NPC (A1 and A2) patient and a NPL patient (B1 and B2); there were overlaps of ADC value distribution between these two cases and their mean values were close (773.04 × 10^−6^ mm^2^/s *vs.* 674.35 × 10^−6^ mm^2^/s). But using a ADC_99_ cut-off value of 1,480 × 10^−6^ mm^2^/s can differ NPC from NPL (1,570 × 10^−6^ mm^2^/s *vs.* 1,210 × 10^−6^ mm^2^/s). It is still possible that ADC histogram will provide more valuable support in a larger and in histologically more specified cohort. Thus, results in our studies can prompt further studies on the role of ADC histogram in the diagnosis of NPL and NPC.

There were several limitations in the present study. First, the sample size of NPL was limited. Further, although these NPL tumors included a variety of pathological types, a statistical analysis among the different pathological types was not performed. Third, only two b-values, 0 and 1,000 s/mm^2^, were set in our DWI sequence, therefore the perfusion effects may bias the ADC values. Further study based on multi-b value DWI sequence is needed. Fourth, further second order texture analysis is needed. Second-order texture parameters, such as entropy, can describe the more subtle aspects of lesion texture, and this would be more valuable for differentiation work.

## Conclusion

In conclusion, the present study indicated that whole-lesion ADC histogram analysis based on high-resolution RESOLVE imaging is effective in differentiating NPL from NPC.

## Data Availability Statement

The raw data supporting the conclusions of this article will be made available by the authors, without undue reservation.

## Ethics Statement

This study was conducted with approval from the Review Committee of the First Affiliated Hospital of Zhengzhou University (No: 2015010039). The patients/participants provided their written informed consent to participate in this study.

## Author Contributions

CS, PC, and SX acquired data. CR, PC, and SX drafted the manuscript. JC and YZ contributed substantially to its revision. All authors take responsibility for the paper as a whole. All authors contributed to the article and approved the submitted version.

## Conflict of Interest

The authors declare that the research was conducted in the absence of any commercial or financial relationships that could be construed as a potential conflict of interest.

## Abbreviations

NPC, Nasopharyngeal carcinoma; NPL, nasopharyngeal lymphoma; MRI, Magnetic resonance imaging; CT, computed tomography; ADC, apparent diffusion coefficient; DWI, diffusion-weighted imaging; ROI, region of interest; ROC, Receiver operating characteristic; SS-EPI, single-shot echo-planar imaging.
